# Draft Genome Sequence of Lignocellulose-Degrading Thermophilic Bacterium *Geobacillus* sp. Strain WSUCF1

**DOI:** 10.1128/genomeA.00595-13

**Published:** 2013-08-15

**Authors:** Aditya Bhalla, Amoldeep Singh Kainth, Rajesh K. Sani

**Affiliations:** Department of Chemical and Biological Engineering, South Dakota School of Mines and Technology, Rapid City, South Dakota, USA

## Abstract

*Geobacillus* sp. strain WSUCF1 is a thermophilic spore-forming member of the phylum *Firmicutes*, isolated from a soil sample collected from the compost facility. We report the draft genome sequence of this isolate with an estimated genome size of 3.4 Mb. The genome sequence of this isolate revealed several genes encoding glycoside hydrolases, making it a potential candidate for plant biomass degradation.

## GENOME ANNOUNCEMENT

Processes utilizing thermophilic lignocellulose-degrading microorganisms have a great potential for the conversion of lignocellulose to biofuels (1, 2). *Geobacillus* sp. strain WSUCF1 (hereafter referred to as WSUCF1) is a thermophilic microbe isolated from a soil sample collected from the compost facility at Washington State University, Pullman, WA, after enrichment on cellulose as a source of carbon and energy. It produces highly thermostable lignocellulose deconstruction enzymes when grown on lignocellulosic substrates, such as corn stover and prairie cord grass (1). In order to reveal the complete gene repertoire of WSUCF1, the genome was sequenced at Integrated Genomics on an Ion Torrent personal genome machine (3). The genome of WSUCF1 is 3,402,383 bp, with a G+C content of 52.21%. The genome was assembled from a total of 2,207,232 sequence reads, with an average read length of 113.65 bp. The reads were assembled into 346 contigs, with an average contig length of about 9,833 bp. The genome contains 4,191 total open reading frames (ORFs) with 47 tRNAs and 3 rRNA operons. The annotations were performed using the ERGO genome analysis suite (4). The results revealed that 17.3% of the total ORFs were assigned for carbohydrate metabolism. Out of 865 ORFs for carbohydrate metabolism, 70 ORFs were found to be involved in polysaccharide degradation. Thirteen predicted open reading frames were annotated as xylan-degrading enzymes and 3 ORFs for cellulose degradation. These results suggest that WSUCF1 holds the potential to be exploited for biofuel production processes. We also identified genes for other industrially important enzymes, such as α-amylases and lipases. The genomic composition of WSUCF1 can be compared to those of other thermophilic and mesophilic organisms to delineate the molecular basis of the thermostability of enzymes.

### Nucleotide sequence accession numbers.

This whole-genome shotgun project has been deposited at DDBJ/EMBL/GenBank under the accession no. ATCO00000000. The version described in this paper is version ATCO01000000.
